# Expression of CD36 by Olfactory Receptor Cells and Its Abundance on the Epithelial Surface in Mice

**DOI:** 10.1371/journal.pone.0133412

**Published:** 2015-07-17

**Authors:** Shinhye Lee, Ai Eguchi, Satoshi Tsuzuki, Shigenobu Matsumura, Kazuo Inoue, Toshihiko Iwanaga, Daisaku Masuda, Shizuya Yamashita, Tohru Fushiki

**Affiliations:** 1 Laboratory of Nutrition Chemistry, Division of Food Science and Biotechnology, Graduate School of Agriculture, Kyoto University, Kyoto, Japan; 2 Laboratory of Histology and Cytology, Graduate School of Medicine, Hokkaido University, Sapporo, Hokkaido, Japan; 3 Department of Cardiovascular Medicine, Osaka University Graduate School of Medicine, Suita, Osaka, Japan; 4 Department of Community Medicine, Osaka University Graduate School of Medicine, Suita, Osaka, Japan; Duke University, UNITED STATES

## Abstract

CD36 is a transmembrane protein that is involved in the recognition of certain amphiphilic molecules such as polar lipids in various tissues and body fluids. So far, CD36 homologues in insects have been demonstrated to be present on the surface of olfactory dendrites and to participate in the perception of exogenous compounds. However, little is known about the relationship between CD36 and mammalian olfaction. Indeed, the detection of only *CD36* mRNA in the mouse olfactory epithelium has been reported to date. In the present study, to provide potential pieces of evidence for the involvement of CD36 in mammalian olfactory perception, we extensively investigated the localisation of this protein in the mouse olfactory mucosa. *In situ* hybridisation analysis using antisense oligonucleotides to *CD36* mRNA detected aggregated signals within the deeper epithelial layer of olfactory mucosa. The mRNA signals were also detected consistently in the superficial layer of the olfactory epithelium, which is occupied by supporting cells. Immunostaining with an anti-CD36 polyclonal antibody revealed that CD36 localises in the somata and dendrites of distinct olfactory receptor cells and that it occurs abundantly on the olfactory epithelial surface. However, immunoreactive CD36 was rarely detectable in the nerve bundles running in the lamina propria of olfactory mucosa, the axons forming the olfactory nerve layer in the outermost layer of the bulb and axon terminals in the glomeruli. We also obtained electron microscopic evidence for the association of CD36 protein with olfactory cilia. Altogether, we suggest that CD36 plays a role in the mammalian olfaction. In addition, signals for CD36 protein were also detected on or around the microvilli of olfactory supporting cells and the cilia of nasal respiratory epithelium, suggesting a role for this protein other than olfaction in the nasal cavity.

## Introduction

CD36 is a transmembrane protein that is expressed in a variety of cells, including monocytes/macrophages, adipocytes, myocytes and taste buds [1, 2]. As suggested by its broad expression pattern, CD36 has a variety of physiological functions, which are associated with its ability to recognise a number of amphiphilic molecules such as polar lipids [1–4]. For instance, CD36 recognises and binds to the oxidised phospholipids of oxidised low-density lipoproteins and mediates the uptake of these particles in macrophages [5, 6]. Another important function of CD36 is to detect and regulate the uptake of long-chain fatty acids (LCFAs) by various cells [7–9]. CD36-knockout mice exhibit an increase in serum-free fatty acid (FA) levels and a reduced uptake of oleate in adipocytes during fasting [8]. Muscle-specific over-expression of CD36 in mice results in enhanced FA oxidation in contracting tissue [9]. Moreover, CD36 appears to play a key role in the gustatory perception of LCFAs, as suggested by its presence in the apical region of rat taste buds [10], a reduction of LCFA palatability in CD36-knockout mice [11] and an LCFA-induced increase in the concentration of intracellular free calcium in taste bud cells expressing CD36 [12].

A family of membrane proteins in insects called sensory neuron membrane proteins (SNMPs) has structural features comparable to those of the class B scavenger receptor family, which includes CD36 [13]. Importantly, some of the SNMPs have been demonstrated to be present on the surface of olfactory dendrites and are involved in the recognition of amphiphilic molecules [14–16]. Despite these studies on SNMPs in insects, few studies have been conducted on the putative function of CD36 in the mammalian olfactory system. To our knowledge, the detection of only *CD36* mRNA in the mouse olfactory epithelium has been reported to date [17]. In the present study, we aimed to confirm CD36 expression in the olfactory epithelium of mice and to determine the location of production and function within the tissue. CD36 was produced by the receptor and supporting cells of the olfactory epithelium in mice, and it is abundant on the epithelial surface containing the olfactory cilia. Furthermore, we also demonstrated the presence of CD36 on the cilia of nasal respiratory epithelium. Our results suggest that CD36 plays role(s) in the olfactory system.

## Materials and Methods

### Animals

This study was approved by the Kyoto University Animal Experimentation Committee (permission number: 26–46), and experiments were conducted in accordance with both the ethics guidelines of the above-mentioned committee and the National Institutes of Health Guide for the Care and Use of Laboratory Animals. CD36-knockout mice were kindly provided by Mason W. Freeman, MD, from Harvard Medical School [18] and maintained on a C57BL6/J background. Wild-type and CD36-knockout littermates were bred from the same cross and were, therefore, of identical genetic background. Animals used in this study were 8–12 weeks old, unless otherwise noted. All process was performed under sodium pentobarbital anesthesia, and all efforts were made to minimize suffering and the number of animals that necessary to produce reliable scientific information.

### Genotyping

Genomic DNA was extracted from the tails of mice. Genotyping was conducted using the GoTaq Hot Start green Master Mix (Promega; Madison, WI, USA) according to the manufacturer’s instructions. Polymerase chain reaction (PCR) was carried out using a PC-708-02 thermal cycler (ASTEC; Fukuoka, Japan). PCR products were separated on a 2% agarose gel, and visualised using SYBR Gold Nucleic Acid Gel Stain (Invitrogen; Carlsbad, CA, USA). For genotyping of the CD36-knockout mice, we used a previously published primer set: 5′-AGCTCCAGCAATGAGCCCAC-3′ (forward for wild-type mice), 5′-TGGAAGGATTGGAGCTACGG-3′ (forward for CD36-knockout mice), and 5′-CATACATTGCTGTTTATGCATGA-3′ (reverse for wild-type and CD36-knockout mice) [19].

### Reverse transcription (RT)-PCR

Total RNA was extracted from the nasal mucosa and liver of mice using the RNeasy Mini Kit (Qiagen; Tokyo, Japan). RT-PCR was performed as described previously [20]. Primer sets (Invitrogen) used were as follows: 5′-GAGCCATCTTTGAGCCTTCA-3′ and 5′-TCAGATCCGAACACAGCGTA-3′ (for CD36); 5′-CCCTGTGCTGCTCACC-3′ and 5′-GCACGATTTCCCTCTCAG-3′ (for β-actin). The amplified products were analysed as described above.

### *In situ* hybridisation analysis

Two non-overlapping antisense oligonucleotide probes (45 bp in length) were designed for the *in situ* detection of mouse *CD36* mRNA. The probes were complementary to the sequence of nucleotides 576 to 620 and 979 to 1023 of mouse *CD36* mRNA (accession number: NM007634), and were labelled with ^33^P-dATP using terminal deoxynucleotidyl transferase (Invitrogen). Fresh-frozen sections of 14-μm thickness were fixed with 4% paraformaldehyde in 0.1 M phosphate buffer for 15 min, and then acetylated with 0.25% acetic anhydride in 0.1 M triethanolamine-HCl (pH 8.0) for 10 min. Hybridisation was performed at 42°C for 10 h with a hybridisation buffer containing the ^33^P-labelled oligonucleotide probes (10,000 cpm/μl). The sections were rinsed in 2× SSC (1× SSC: 150 mM sodium chloride, 15 mM sodium citrate) containing 0.1% *N*-lauroylsarcosine sodium at room temperature for 30 min, and then rinsed twice in 0.1× SSC containing 0.1% *N*-lauroylsarcosine sodium at 55°C for 40 min. The sections were dehydrated through a graded series of ethanol and air-dried. Sections were immersed in an autoradiographic emulsion (NTB-2; Kodak, Rochester, NY, USA) at 4°C for 8–10 weeks. After development, the hybridised sections were counterstained with hematoxylin. The specificity of the hybridisation was confirmed by the absence of signal upon the addition of excess amounts of unlabelled antisense oligonucleotide probe.

### Western blot analysis

Tissue pieces were lysed by sonication in a lysis buffer (50 mM Tris-HCl (pH 8.0), 150 mM NaCl, 0.5% NP-40) containing protease inhibitor cocktail (P8340, Sigma-Aldrich; St. Louis, MO, USA) and phosphatase inhibitor cocktail (P0044, Sigma-Aldrich). The protein concentrations in the tissue lysates were determined using the bicinchoninic acid protein assay kit (Pierce Chemical; Rockford, IL, USA). The lysates (40 μg of protein) and a molecular weight standard (MagicMark XP, Invitrogen) were subjected to sodium dodecyl sulphate-polyacrylamide gel electrophoresis (SDS-PAGE, on a 15% gel) under reducing conditions and transferred to a polyvinylidene difluoride membrane. After blocking with Tris-buffered saline (pH 7.4) containing 5% skim milk and 0.1% Tween-20 for 1 h, the membrane was incubated overnight at 4°C with a goat polyclonal anti-CD36 antibody (1:2000; AF2519; R&D Systems, Minnesota, MN, USA). After several washes, peroxidase-labelled polyclonal rabbit anti-goat IgG secondary antibody (1:1000; P0449; Dako; Tokyo, Japan) was added. Immunolabelled protein complexes were detected using an ImageQuant LAS-4000 mini chemiluminescent imager (GE Healthcare; Tokyo, Japan).

### Immunohistochemistry

The mice were anaesthetised with sodium pentobarbital (10 mg/kg body weight) and were perfused via the aorta with saline (0.5 ml/g) followed by a fixative (4% paraformaldehyde in 0.1 M phosphate buffer, pH 7.4). The heads and tongues were removed and immersed in the same fixative for an additional 2 days. For decalcification, the heads were immersed in 5% ethylenediaminetetraacetic acid for 3 weeks at 4°C. The decalcified heads were immersed in 30% sucrose solution overnight at 4°C, embedded in optimal cutting temperature compound (Sakura Finetek; Tokyo, Japan), and quickly frozen in liquid nitrogen. Frozen sections were mounted on poly-L-lysine-coated glass slides. After pre-treatment with phosphate-buffered saline (PBS) containing 0.3% Triton X-100 and normal donkey serum, the sections were incubated overnight with AF2519 (1:2000). The sections were then incubated with Cy3-labelled donkey polyclonal anti-goat IgG (Jackson ImmunoResearch; 1: 200; 705-165-147; West Grove, PA, USA). The nuclei were counterstained with SYTO 13 (Invitrogen). For double immunofluorescence staining, the sections were incubated overnight with AF2519 and the following antibodies; rabbit polyclonal antibody to olfactory marker protein (OMP) (anti-OMP; 1:1000; O7889; Sigma-Aldrich), rabbit polycolonal antibody to PGP9.5 (anti-PGP9.5; 1:2000; RA95101; UltraClone Ltd, Rossiters, UK) and rabbit polyclonal antibody to S100 protein (anti-S100; RY320: 1:4000; Yanaihara Institute, Fujinomiya, Japan). Then, they were incubated with Cy3-labelled donkey anti-goat IgG and Alexa Fluor 488-labelled donkey anti-rabbit IgG (Invitrogen). The stained sections were mounted in glycerol/PBS and observed under a confocal laser-scanning microscope (FV1000; Olympus; Tokyo, Japan).

### Silver-intensified immunogold labelling method for electron microscopy

Frozen sections were incubated overnight with anti-CD36 antibody (1 μg/ml) and, subsequently, with rabbit anti-goat IgG covalently linked with 1-nm gold particles (1:200; Nanoprobes; Yaphank, NY, USA). Following silver enhancement using the HQ SILVER enhancement kit (Nanoprobes), the sections were osmicated, dehydrated and directly embedded in Epon (Nisshin EM; Tokyo, Japan). Ultrathin sections were stained with uranyl acetate and lead citrate, and examined under an electron microscope (H-7100; Hitachi; Tokyo, Japan).

## Results

### RT-PCR and *in situ* hybridisation analyses of CD36 in the nasal mucosa of mice

**Fig 1:**
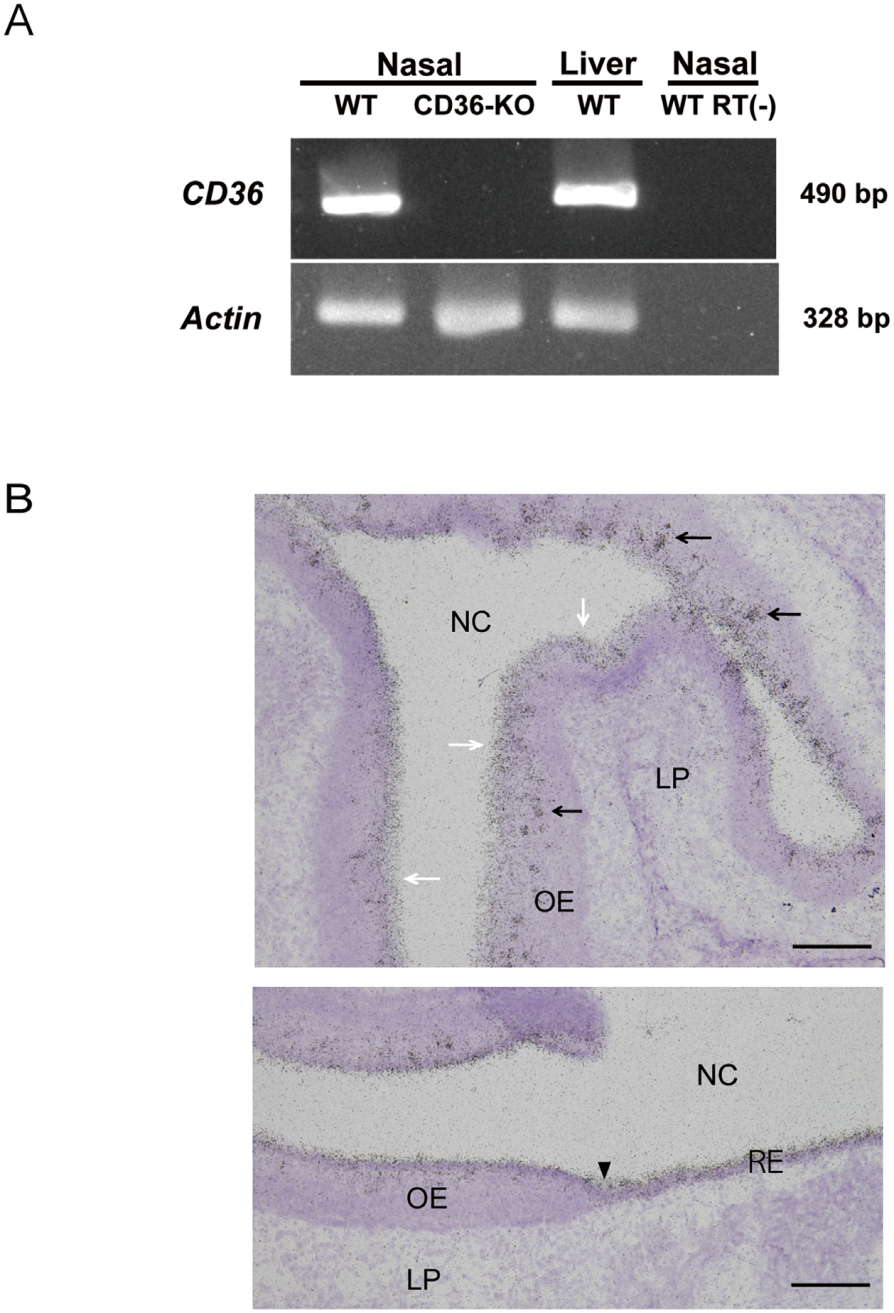
Detection of *CD36* mRNAs in the nasal mucosa of mice. (A) RT-PCR detection. RT-PCR was conducted as described in the Materials and Methods section using nasal samples of wild-type (WT) and CD36-knockout mice (CD36-KO). RT(—) indicates the reaction without reverse transcriptase. (B) *In situ* hybridisation analysis of the nasal mucosa of wild-type mice. The upper and lower panels show the data of olfactory mucosa and the transitional zone to the respiratory area, respectively. Aggregated signals are distributed within the epithelial layer (black arrows in the upper panel). The signals were also detected consistently in the superficial layer of the olfactory epithelium (white arrows in the upper panel). A black arrowhead in the lower panel indicates the boundary between the olfactory (left-hand side) and respiratory (right-hand side) mucosa. These pictures are representatives of those from six different sections obtained using two animals. LP, lamina propria; NC, nasal cavity; OE, olfactory epithelium; RE, nasal respiratory epithelium. Bar: 100 μm (upper panel), 50 μm (lower panel).

CD36 expression in the olfactory mucosa of mice has previously been demonstrated using RT-PCR and *in situ* hybridisation methods [17]. In this study, we examined RT-PCR detection of *CD36* mRNA in the nasal mucosa of wild-type and CD36-knockout mice. The β-actin gene *Actb* was evaluated as a control for RNA degradation. The expression of both genes in the liver was examined as a reference. PCR products for both *CD36* (490 bp) and *Actb* (328 bp) were detected in the nasal mucosa and in the liver samples of wild-type mice (Fig 1A). In the nasal mucosa samples of CD36-knockout mice, amplicons of *Actb*, but not of *CD36*, were detected (Fig 1A). In control RT-PCR mixtures prepared without reverse transcriptase, neither *CD36* nor *Actb* products were detected (Fig 1A).

Next, we aimed to detect *CD36* mRNA in the olfactory mucosa by using *in situ* hybridisation with ^33^P-labelled oligonucleotides. When sections of the olfactory mucosa were probed with an antisense oligonucleotide directed against *CD36* mRNA (576–620), aggregated signals were detected within the epithelial layer (Fig 1B, black arrows in the upper panel). This observation is consistent with previously reported results [17]. However, unlike previous results [17], signals were also detected consistently in the superficial layer of the olfactory epithelium, which is occupied by supporting cells (Fig 1B, white arrows in the upper panel). In addition, *CD36* mRNA expression was detected in the superficial layer throughout the respiratory area (Fig 1B, a black arrowhead on the lower panel indicates the boundary between olfactory and respiratory areas). Identical results were obtained using another oligonucleotide (S1 Fig).

Sections of whole-head preparations from wild-type and CD36-knockout mice were subjected to *in situ* hybridisation with the probe for *CD36* mRNA (576–620). Hybridisation signals were detected in the distinct areas of the section obtained from a wild-type mouse, including those of nasal mucosa, sebaceous gland and tongue, whereas no significant ones could be seen throughout the section from a CD36-knockout mouse (S2 Fig). These results support the validity of our *in situ* detection of *CD36* mRNA.

### Western blot analysis of CD36 expression in the nasal mucosa of mice

**Fig 2:**
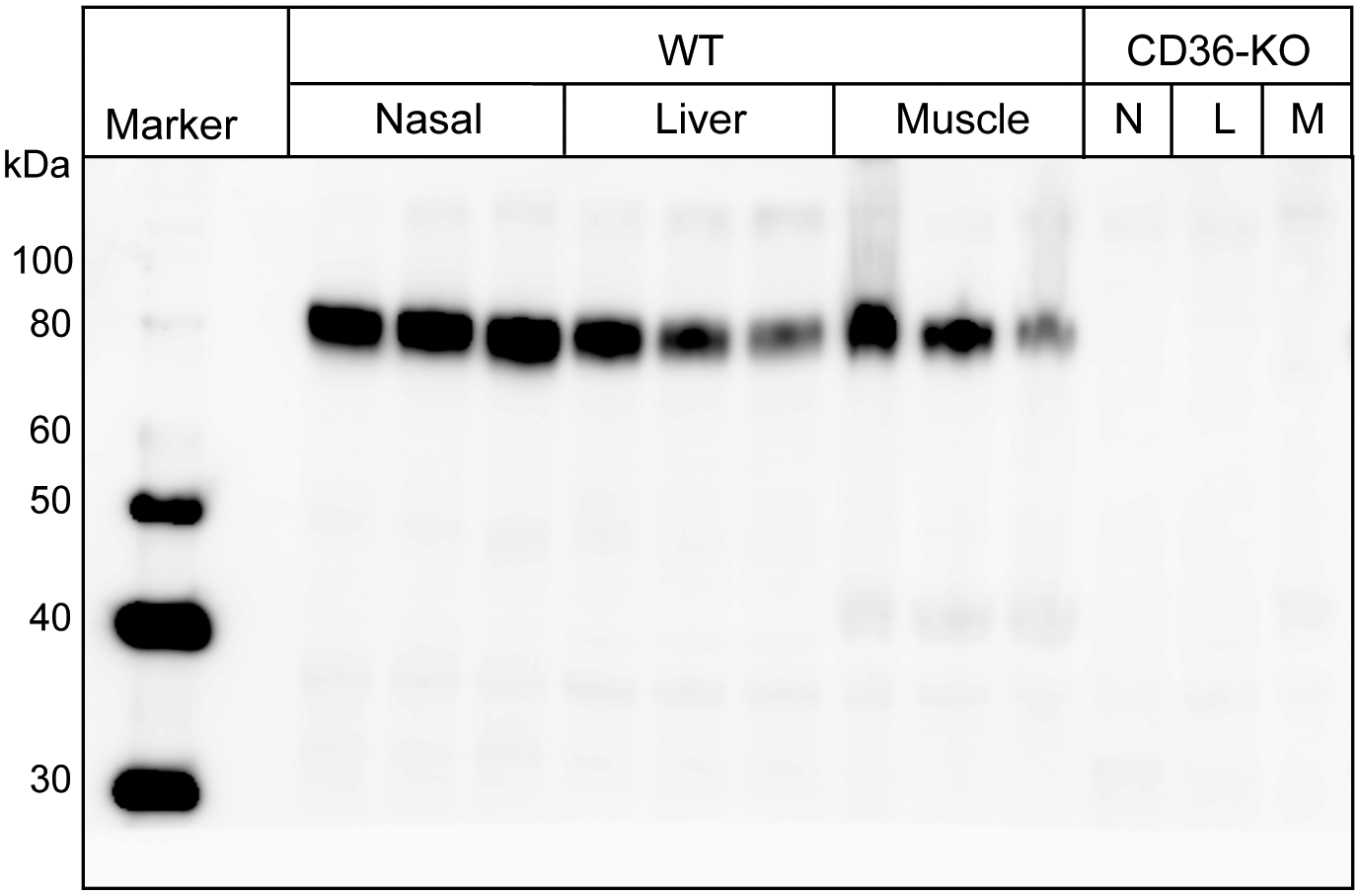
Western blot analysis of lysates from the nasal mucosa (and other tissues) of mice. Lysates obtained from the nasal mucosa (Nasal), liver and skeletal muscle (Muscle) of wild-type mice (WT) and those (N; nasal mucosa, L; liver, M; skeletal muscle) of CD36-knockout mice (CD36-KO) were analysed by SDS-PAGE and western blotting with AF2519. Samples from wild-type mice were analysed in triplicate. The molecular sizes of the protein marker (Marker) are indicated on the left in kilodaltons (kDa).

CD36 protein expression in the nasal mucosa of wild-type mice was analysed by SDS-PAGE and western blotting. Lysates of liver and skeletal muscle (tissues for CD36 expression) were subjected to the analysis simultaneously with that of nasal mucosa for a qualitative assessment of the resulting data. Various anti-CD36 antibodies were tested, but only one (AF2519) produced the expected 88-kDa band. The band was detected in the nasal mucosa as well as in the liver and skeletal muscle samples (Fig 2). Note that the level of the signal in the lysate of nasal mucosa was comparable (or even superior) to that in those of the other tissues. Nasal mucosa, liver and skeletal muscle lysates from CD36-knockout mice did not yield apparent protein bands around the 88-kDa position with the anti-CD36 antibody (Fig 2).

### Immunolocalisation of CD36 in the olfactory mucosa of mice

**Fig 3:**
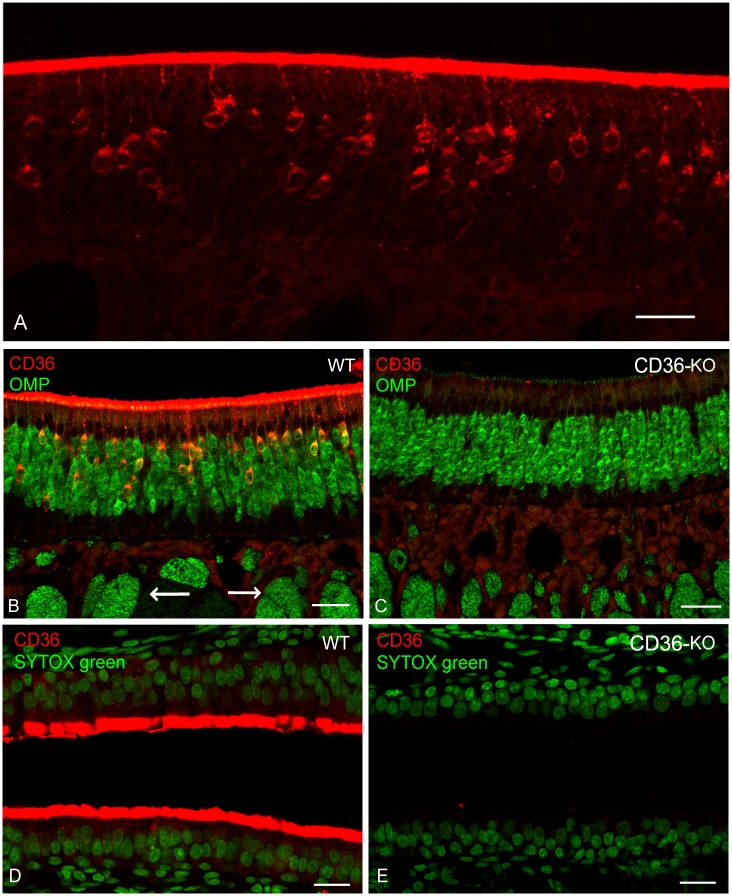
Immunolocalisation of CD36 in the mouse nasal mucosa. (A) Immunostaining of the olfactory mucosa of wild-type mice with AF2519. Positive immunoreactivity for CD36 is seen in cells with thin dendrites and in the surface layer. (B, C) Double immunostaining of olfactory mucosa with AF2519 (red) and an anti-OMP antibody (green) of olfactory mucosa in wild-type (WT) and CD36-knockout (CD36-KO) mice. In (B), bundles of nerve fibres in the lamina propria are indicated by white arrows. (D, E) Immunostaining of respiratory mucosa of wild-type (WT) and CD36-knockout mice (CD36-KO) with AF2519. The red-coloured immunoreactivity for CD36 in the surface layer was not evident in CD36-knockout mice. In (D) and (E), nuclei are labelled with SYTOX (green). Each picture is a representative of those from at least three different sections. In each immunostaining analysis, sections were obtained using at least two animals. Each of the pictures is a representative of those from at least three different sections. Bar: 100 μm (A), 50 μm (B, C, D and E).

The main purpose of this study was to localise CD36 protein in the olfactory mucosa. In this study, we used AF2519 as the probe for immunohistochemical localisation of CD36. When the olfactory mucosa of wild-type mice was probed with the antibody, some but not all cells in the deeper epithelial layer were stained (Fig 3A). The cells extended a slender process to the epithelial surface passing through the supporting cell layer, thus, being identifiable as olfactory receptor cells (ORCs). In addition, the surface layer of the epithelium was found to be heavily stained (Fig 3A).

We performed double immunostaining of the olfactory mucosa of wild-type mice with AF2519 and an antibody against OMP (a marker protein for ORCs), and found that CD36-immunoreactive cells within the epithelial layer stained positive with anti-OMP antibody (Fig 3B), further confirming the production of CD36 by ORCs. However, the CD36 antibody failed to stain the olfactory nerves of various sizes running in the lamina propria (Fig 3B, white arrows). Accordingly, olfactory nerve elements, which were stained with anti-OMP antibody, were immunonegative with AF2519 in the olfactory bulb (S3 Fig). When olfactory mucosa from CD36-knockout mice was stained, immunoreactivity with AF2519 was not visible in either the ORCs or the surface layer, while the staining pattern by the anti-OMP antibody was similar to that observed in wild-type mice (Fig 3C), confirming the validity of our immunolocalisation of CD36 in wild-type mice. In the context of olfactory mucosa of wild-type mice, double immunostaining with AF2519 and an antibody against a pan-neuronal marker, PGP9.5, gave similar results to those with AF2519 and anti-OMP antibody (S4A Fig). In double immunostaining of the olfactory mucosa of wild-type mice with AF2519 and an antibody raised against a glial marker S100, signals for CD36 protein were still undetectable on the bundles running in the lamina propria where glial (labelled in a green colour) and neuronal elements coexist (S4B Fig).

We also performed immunostaining with AF2519 of the nasal respiratory mucosa and the site of pheromone detection, vomeronasal organ (VNO), of wild-type and CD36-knockout mice. Staining of the surface layer in the nasal respiratory area with the antibody was evident in wild-type mice but not in CD36-knockout mice (Fig 3D and 3E, respectively). We could not detect significant signal for CD36 in the sensory epithelium of VNO in either wild-type or CD36-knockout mice (S5 Fig).

### Immunoelectron microscopic analysis of CD36 localisation in the mouse nasal mucosa

**Fig 4:**
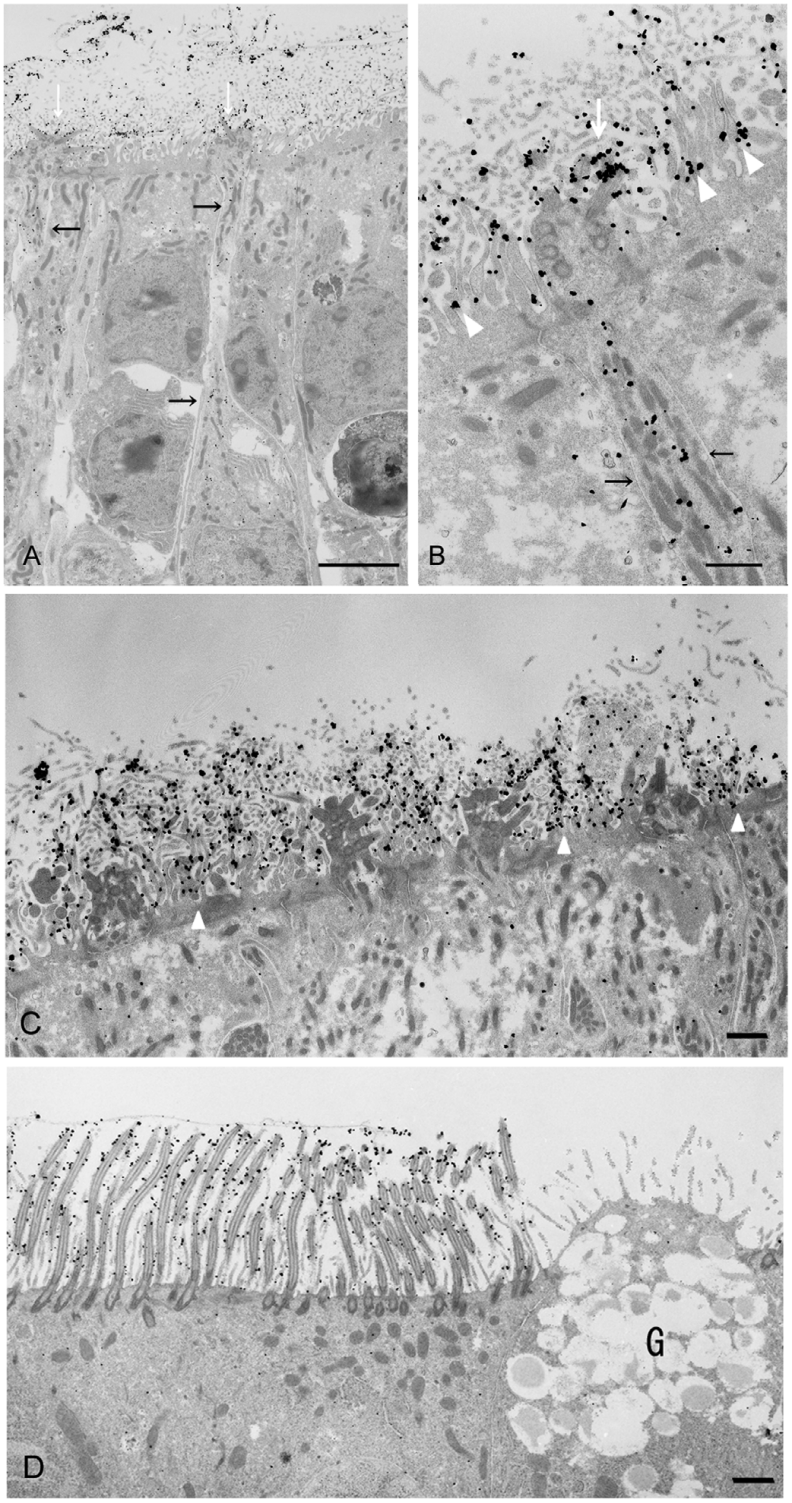
Immunoelectron microscopic analysis of CD36 localisation in the nasal mucosa of wild-type mice. Immunoelectron microscopic analysis of the nasal olfactory (A, B and C) and respiratory (D) epithelium. Dotted particles showing the existence of CD36 distribute in the somata and dendrites of ORCs (black arrows in A and B). The particles on the olfactory cilia and on or around the microvilli of supporting cells are indicated by white arrows (A and B) and white arrowheads (B and C), respectively. These pictures are representatives of those from more than thirty sections obtained using three animals. G: goblet cells. Bar: 5 μm (A), 1 μm (B, C, D).

We further analysed the localisation of CD36 in the olfactory and respiratory areas of nasal mucosa by immunoelectron microscopy with AF2519 and a colloidal gold-labelled anti-goat IgG antibody. Representative data for the olfactory epithelium of wild-type mice are presented in Fig 4A, 4B and 4C. Immunogold particles were evident in the soma and dendrites of distinct ORCs within the epithelium (black arrows in A and B). In the surface layer, the particles heavily labelled the olfactory cilia (white arrows in A and B) and also attached to the microvilli of olfactory supporting cells (white arrowheads in B). Electron microscopic images within which no or few dotted particles were visible on ORCs more clearly illustrated the localisation of signals on the microvilli of supporting cells (white arrowheads in C). Representative data for the respiratory epithelium of wild-type mice are presented in Fig 4D. Gold particles were evident in the cilia of respiratory ciliary cells. On the other hand, very few immunogold particles were detected in the somata of supporting cells of olfactory epithelium (Fig 4A, 4B and 4C) and ciliary cells of respiratory epithelium (Fig 4D).

## Discussion

Our aim was to localise the site(s) of production and function of CD36 in the murine olfactory epithelium. In the present study, using *in situ* hybridisation and immunohistochemical analyses, we provided compelling evidence for the production of CD36 by ORCs in the mouse olfactory epithelium. Strikingly, an intense immunoreactivity for CD36 was observed in the surface layer throughout the olfactory epithelium (Fig 3A). It seemed that these surface signals were, at least in part, due to CD36 protein produced in ORCs and transported to the cilia, as immunogold particles were present in the dendrites and somata of ORCs and cilia (Fig 4A and 4B). Altogether, we propose that a major site of function for CD36 in the olfactory mucosa is on the cilia.

Contrary to the epithelial layer, CD36 was rarely detectable in the nerve bundles running in the lamina propria of olfactory mucosa, the axons forming the olfactory nerve layer in the outermost layer of the bulb and axon terminals in the glomeruli (S3 and S4 Figs). The CD36 localisation pattern differs from that of G-protein-coupled odorant receptors, which are often observed in the axon termini as well as in somata, dendrites, and cilia [21, 22]. However, the CD36 localisation pattern resembles that of certain SNMPs in the invertebrate olfactory organs. For example, Snmp-1 protein in the antenna of the silk moth localises to the cilia, dendrites and somata, but rarely to the axons or nerve bundles [14, 16]. Importantly, CD36-related protein has been found to co-localise with G-protein-coupled odorant receptors and is postulated to act as a co-receptor [15]. These findings, together with the specific immunolabelling pattern in distinct ORCs observed in our study, led us to speculate that CD36 is produced only by ORCs expressing certain types of odorant receptors, and that it plays a supportive role for these receptors.

Certain SNMPs have been found to serve as co-receptors for distinct types of pheromone receptors [15]. Furthermore, in the mouse brain, *CD36* mRNA has been found to express in the regions involved in reproductive behaviour (e.g., bed nucleus of stria terminalis), which is thought to primarily rely upon the detection of pheromone by VNO [23]. These previous findings lead to the hypothesis that CD36 could play a role in the detection and/or delivery of pheromones in specific neural circuits of mice. However, CD36 does not appear to serve to detect pheromones at the VNO of mice. Indeed, no significant signals for CD36 were detected in the sensory epithelium of VNO (S5 Fig). Reproductive behaviours in mice are also known to be affected by odorants detected through main olfactory epithelium [24]. Involvement and role of CD36 in the murine reproductive behaviours await further investigation.

We detected *CD36* mRNA expression consistently in the superficial cell layer of the olfactory epithelium, where the supporting cells are localised (Fig 1B and S1A Fig). However, signal for CD36 protein was detected only on or around microvilli of the supporting cells (Fig 4B and 4C). These findings, together with the intense signals observed throughout the surface layer of the olfactory epithelium (Fig 3A) led to the assumption that a number of CD36 molecules produced in olfactory supporting cells are released into the extracellular milieu (e.g., into the mucous layer of the olfactory epithelium). Indeed, CD36 is known to exist in the plasma fraction of blood, as a constituent of microparticles derived from platelets and other cells [25]. The destiny of CD36 in the olfactory supporting cells, including the formation of CD36-containing microparticles from the cells, awaits further investigation. We also detected *CD36* mRNA in the superficial cell layer of the nasal respiratory epithelium (Fig 1B and S1B Fig), and revealed many immunogold particles attached to cilia of the ciliary cells (Fig 4D). Certain SNMPs have been shown to be located in non-neuronal epithelial cells and in supporting cells around the olfactory sensory neurons [15, 16]. Heliothis virescens SNMP-2 is assumed to be involved in quick pheromone clearance of the cuticular sensilla lymph to allow for highly sensitive olfactory detection [26]. By analogy, we postulated that CD36 expressed by the olfactory supporting cells and respiratory ciliary cells contribute to the clearance of noxious substances such as oxidised phospholipids present in the nasal cavity.

In summary, our study provides evidence that CD36 is produced by ORCs and that it occurs abundantly in the superficial layers of the olfactory epithelium. The site of production and the localisation, together with its ability to recognise a variety of amphiphilic molecules, lead to the hypothesis that CD36 is intimately involved in olfactory reception. In addition, CD36 expression in olfactory supporting cells and nasal respiratory ciliary cells suggests additional role(s) for this protein in the nasal cavity. Further studies are required to clarify the importance of CD36 in the olfactory system.

## Supporting Information

S1 Fig *In situ* hybridisation analysis of the nasal olfactory (A) and respiratory (B) mucosa of wild-type mice with a ^33^P-labelled antisense oligonucleotide directed against *CD36* mRNA (979–1023) as a probe. These pictures are representatives of those from six different sections obtained using two animals. Bar: 50 μm.
(TIF)

S2 Fig *In situ* hybridisation analysis of sections of whole-head preparations from wild-type (WT) and CD36-knockout mice (CD36-KO) with ^33^P-labelled antisense oligonucleotide directed against *CD36* mRNA (576–620) as a probe. Sections were obtained using one each of wild-type and CD36-knockout littermates at postnatal day 5. Signals were detected by autoradiography on X-ray film. The picture is a representative of those from three different sections.
(TIF)

S3 Fig Double immunostaining of the olfactory bulb from wild-type (WT) and CD36-knockout mice (CD36-KO) with AF2519 (red) and anti-OMP antibody (green). Axons forming the olfactory nerve layer (ONL) and glomeruli (GL) were negative for staining with AF2519. Sections were obtained using two each of wild-type and CD36-knockout littermates. Each of the pictures is a representative of those from at least three different sections. Bar: 20 μm (A, B).
(TIF)

S4 Fig Double immunostaining of the olfactory mucosa of wild-type mice with AF2519 (red) and anti-PGP9.5 antibody (green) or anti-S100 antibody (green). The red-coloured immunoreactivity for CD36 occurred in some ORCs but not evident in nerve bundles running in the lamina propria. Neuronal and glial cells are labelled green with antibodies for PGP 9.5 and S100, respectively. Sections were obtained using two animals. Each of the pictures is a representative of those from at least three different sections. Bar: 20 μm (A, B).
(TIF)

S5 Fig Immunostaining of mouse VNO with AF2519. The red-coloured immunoreactivity for CD36 was not evident in either wild-type (WT) or CD36-knockout mice (CD36-KO). In both panels, nuclei are labelled with SYTOX (green). Sections were obtained using two each of wild-type and CD36-knockout littermates. Each of the pictures is a representative of those from at least three different sections. Bar: 20 μm.
(TIF)
