# Supplementary figures and images for: Expression of CD36 by Olfactory Receptor Cells and Its Abundance on the Epithelial Surface in Mice
Source: PLoS One. 2015 Jul 17;10(7):e0133412. doi: 10.1371/journal.pone.0133412 (PMC4506127; doi:10.1371/journal.pone.0133412)

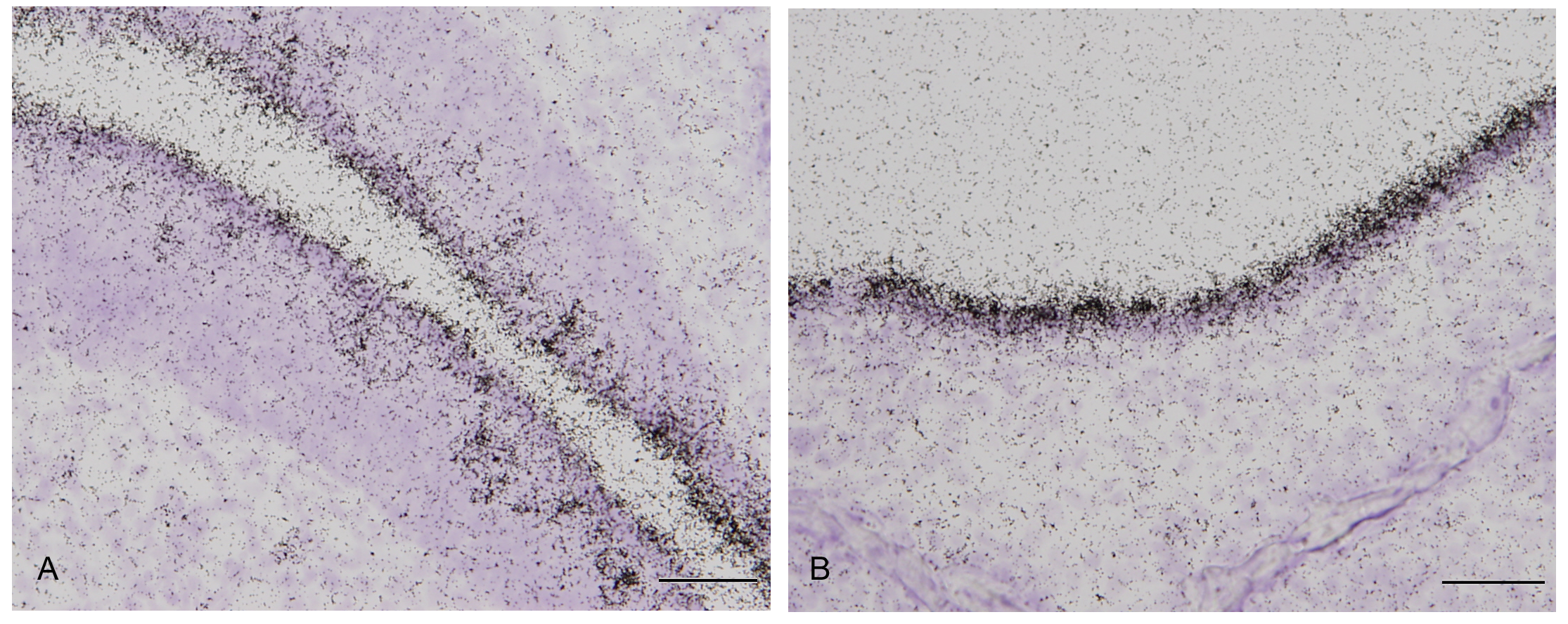

Supplement: S1 Fig — These pictures are representatives of those from six different sections obtained using two animals. Bar: 50 μm. (TIF) [file pone.0133412.s001.tif]

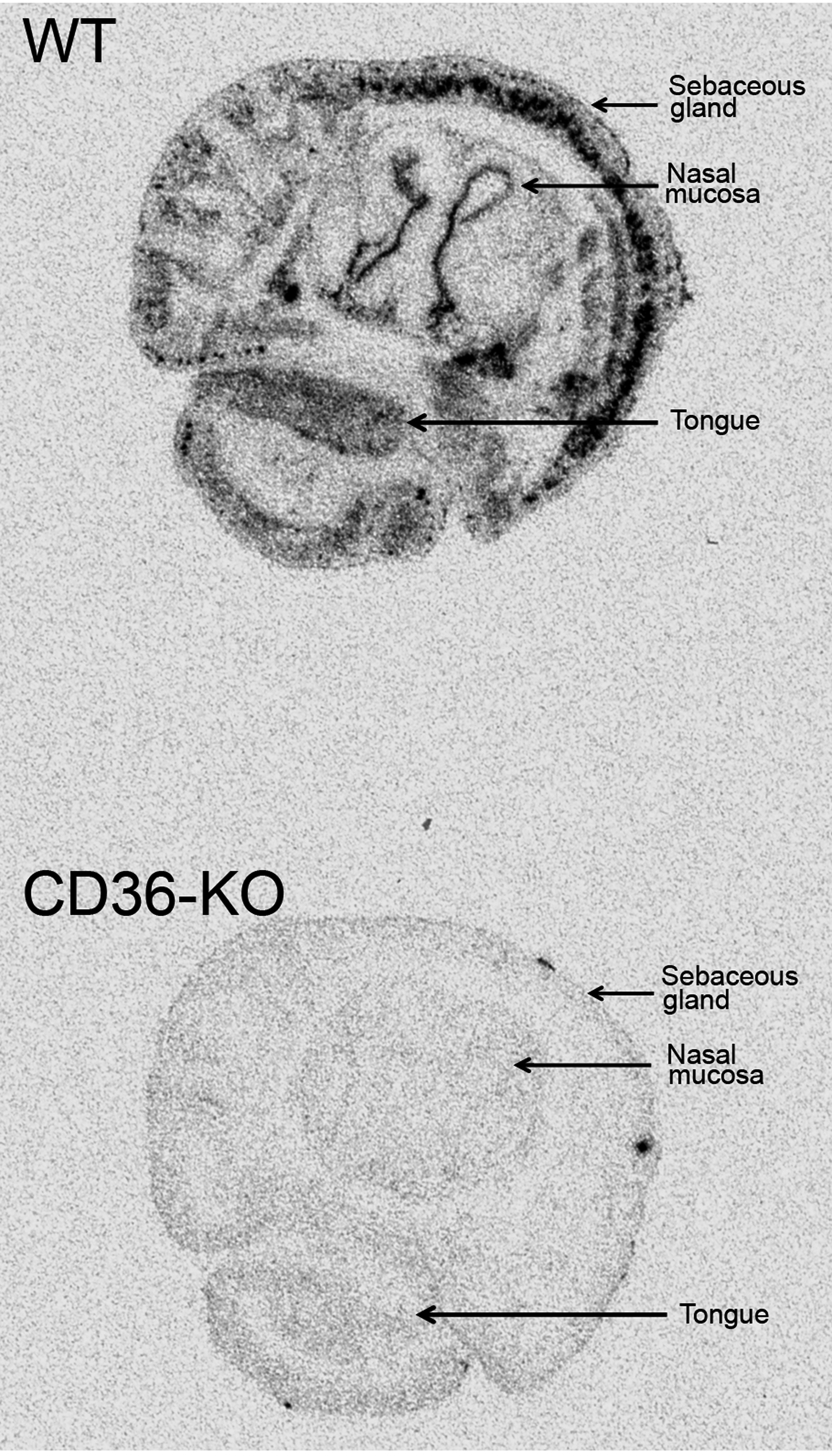

Supplement: S2 Fig — Sections were obtained using one each of wild-type and CD36-knockout littermates at postnatal day 5. Signals were detected by autoradiography on X-ray film. The picture is a representative of those from three different sections. (TIF) [file pone.0133412.s002.tif]

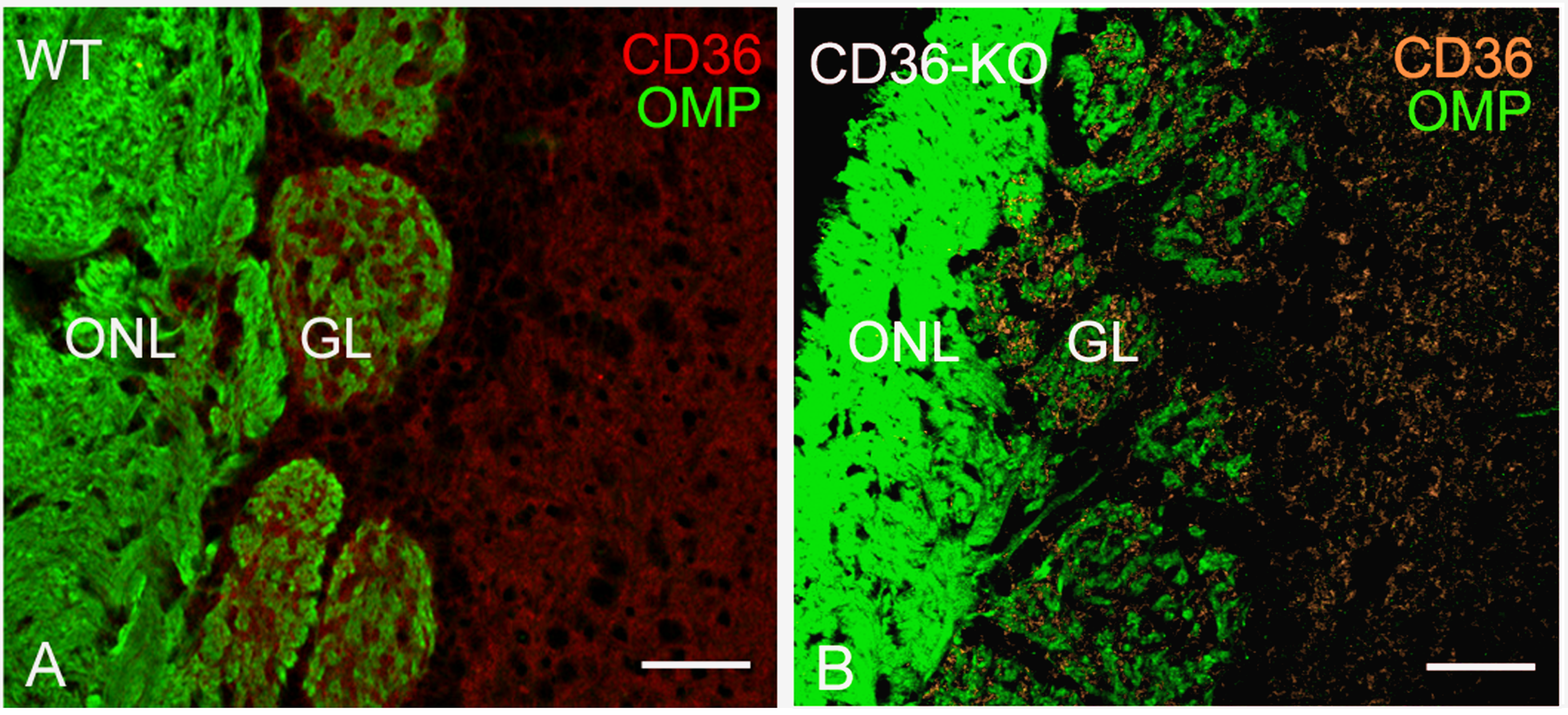

Supplement: S3 Fig — Axons forming the olfactory nerve layer (ONL) and glomeruli (GL) were negative for staining with AF2519. Sections were obtained using two each of wild-type and CD36-knockout littermates. Each of the pictures is a representative of those from at least three different sections. Bar: 20 μm (A, B). (TIF) [file pone.0133412.s003.tif]

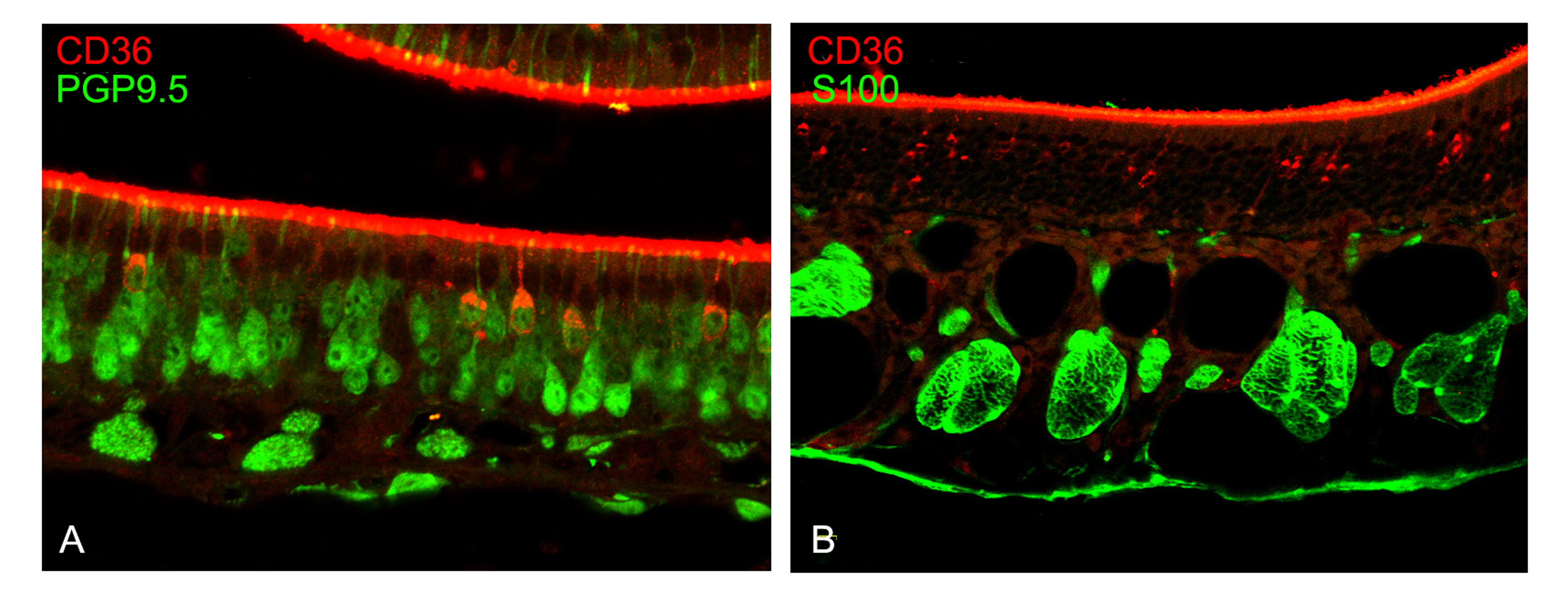

Supplement: S4 Fig — The red-coloured immunoreactivity for CD36 occurred in some ORCs but not evident in nerve bundles running in the lamina propria. Neuronal and glial cells are labelled green with antibodies for PGP 9.5 and S100, respectively. Sections were obtained using two animals. Each of the pictures is a representative of those from at least three different sections. Bar: 20 μm (A, B). (TIF) [file pone.0133412.s004.tif]

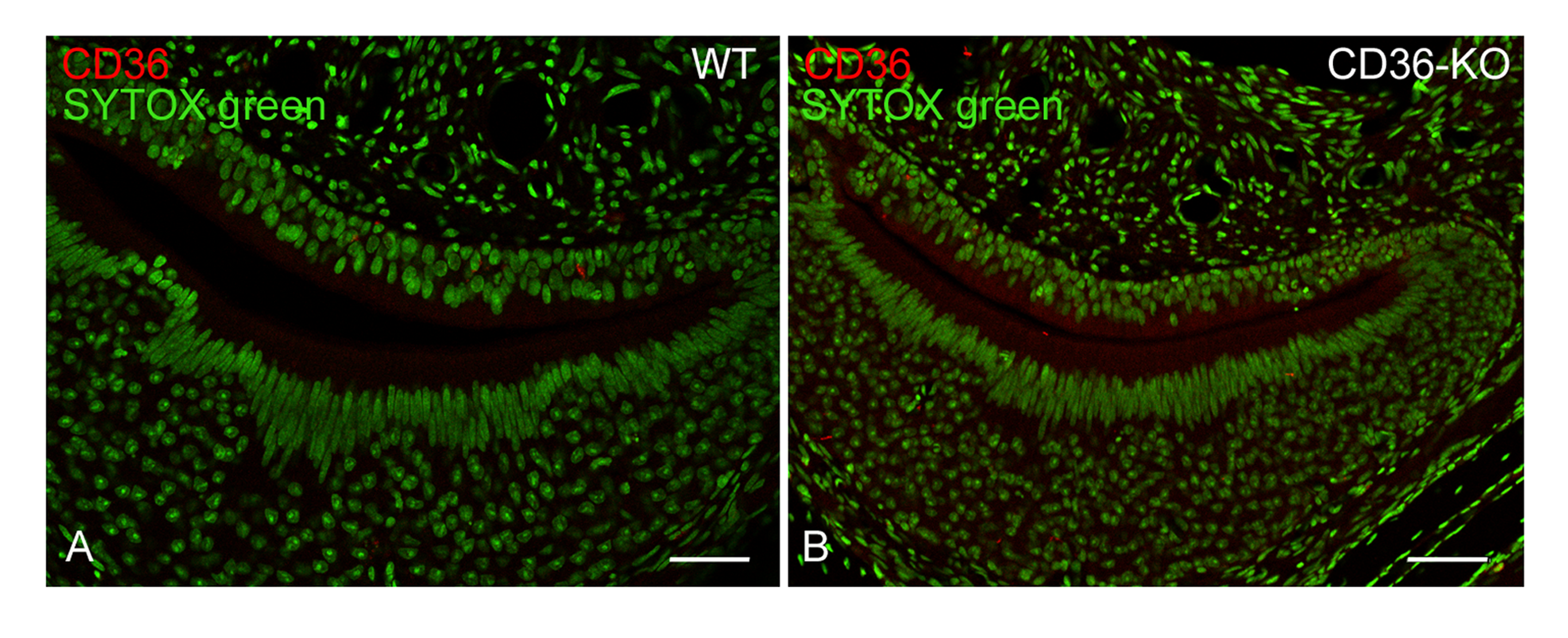

Supplement: S5 Fig — The red-coloured immunoreactivity for CD36 was not evident in either wild-type (WT) or CD36-knockout mice (CD36-KO). In both panels, nuclei are labelled with SYTOX (green). Sections were obtained using two each of wild-type and CD36-knockout littermates. Each of the pictures is a representative of those from at least three different sections. Bar: 20 μm. (TIF) [file pone.0133412.s005.tif]
